# The role of cohort studies for NCD prevention and health promotion – the example of physical activity

**DOI:** 10.1186/1753-6561-7-S5-O14

**Published:** 2013-08-30

**Authors:** Brian Martin

**Affiliations:** 1 University of Zürich, Switzerland

## 

There has been transition in the profile of health risks from traditional risks such as under nutrition and low sanitation towards modern risks such as obesity and overweight. This has resulted in a high burden of non-communicable diseases such as diabetes, cardiovascular diseases, cancers and chronic respiratory diseases. Cross sectional studies, case-control studies and randomised controlled trials have significant challenges in exposure assessments and studying their long-term effects with respect to non-communicable diseases. Cohort studies are ideal for the study of long term effects on NCD outcomes, to study temporal sequence of exposure and potential outcomes, to study changes in exposure and may allow nested case-control studies with prospective exposure assessment.

Physical activity plays an important role in non-communicable diseases and despite the initial success, more evidence based advocacy is necessary. There are concerns whether the resolutions of the UN NCD summit can be implemented speedily and physical inactivity is not included in the first set of targets currently under discussion for WHO's monitoring framework. Better methods for quantification of levels of physical activity and assessment of different domains of physical activity are now available and more trans-cultural research in physical activity and health is necessary. Indo-Swiss partnership in this area assumes importance in the above context.

